# Correction: Chen, X.; Liao, Y. Piezoelectric and Dielectric Properties in Bi_0.5_(Na,K)_0.5_TiO_3_-x Ag_2_O Lead-Free Piezoceramics. *Materials* 2023, *16*, 5342

**DOI:** 10.3390/ma19102138

**Published:** 2026-05-20

**Authors:** Xiaoming Chen, Yunwen Liao

**Affiliations:** 1 School of Materials and Energy Engineering, Guizhou Institute of Technology, Guiyang 550003, China; 2 College of Chemistry and Engineering, China West Normal University, Nanchong 637009, China; liao-yw@163.com

In the original publication [1], the citation referring to reference 4 in the manuscript has been verified by academic editors to be irrelevant. Concerns were raised on this matter, and the following reference was removed from the reference list:

4. Craciun, E.M.; Rabaea, A.; Das, S. Cracks Interaction in a Pre-Stressed and Pre-Polarized Piezoelectric Material. *J. Mech.* **2020**, *36*, 177–182.

With this correction, the order of some references has been adjusted accordingly. The authors state that the scientific conclusions are unaffected. This correction was approved by the Academic Editor. The original publication has also been updated.
